# A pediatric near-infrared spectroscopy brain-computer interface based on the detection of emotional valence

**DOI:** 10.3389/fnhum.2022.938708

**Published:** 2022-09-23

**Authors:** Erica D. Floreani, Silvia Orlandi, Tom Chau

**Affiliations:** ^1^Bloorview Research Institute, Holland Bloorview Kids Rehabilitation Hospital, Toronto, ON, Canada; ^2^Institute of Biomedical Engineering, University of Toronto, Toronto, ON, Canada; ^3^Department of Biomedical Engineering, University of Bologna, Bologna, Italy

**Keywords:** pediatric, affective state, neurofeedback, alternative and augmentative communication, brain-computer interface, functional near-infrared spectroscopy

## Abstract

Brain-computer interfaces (BCIs) are being investigated as an access pathway to communication for individuals with physical disabilities, as the technology obviates the need for voluntary motor control. However, to date, minimal research has investigated the use of BCIs for children. Traditional BCI communication paradigms may be suboptimal given that children with physical disabilities may face delays in cognitive development and acquisition of literacy skills. Instead, in this study we explored emotional state as an alternative access pathway to communication. We developed a pediatric BCI to identify positive and negative emotional states from changes in hemodynamic activity of the prefrontal cortex (PFC). To train and test the BCI, 10 neurotypical children aged 8–14 underwent a series of emotion-induction trials over four experimental sessions (one offline, three online) while their brain activity was measured with functional near-infrared spectroscopy (fNIRS). Visual neurofeedback was used to assist participants in regulating their emotional states and modulating their hemodynamic activity in response to the affective stimuli. Child-specific linear discriminant classifiers were trained on cumulatively available data from previous sessions and adaptively updated throughout each session. Average online valence classification exceeded chance across participants by the last two online sessions (with 7 and 8 of the 10 participants performing better than chance, respectively, in Sessions 3 and 4). There was a small significant positive correlation with online BCI performance and age, suggesting older participants were more successful at regulating their emotional state and/or brain activity. Variability was seen across participants in regards to BCI performance, hemodynamic response, and discriminatory features and channels. Retrospective offline analyses yielded accuracies comparable to those reported in adult affective BCI studies using fNIRS. Affective fNIRS-BCIs appear to be feasible for school-aged children, but to further gauge the practical potential of this type of BCI, replication with more training sessions, larger sample sizes, and end-users with disabilities is necessary.

## 1. Introduction

Early intervention with alternative and augmentative communication (AAC) is crucial to mitigate challenges faced by children with complex communication needs in their cognitive, social, and educational development (Romski et al., 2015). However, for children with cerebral palsy, neurodegenerative disorders or traumatic brain injuries, severe motor impairments can affect their ability to access AAC devices (Tai et al., 2008). Most current alternate access options (i.e., mechanical and proximity switches, adapted trackpads) still require some degree of voluntary physical movement, making them fatiguing or unreliable for those with significant disabilities (Myrden et al., 2014). More recent advances in alternate access technologies, such as eye gaze devices, head trackers, and custom sensors for detecting reproducible patterns in limb movements or vocalizations, are not yet reliable across a wide range of environments and situations (Myrden et al., 2014).

Recently, brain-computer interfaces (BCIs) have attracted attention as an AAC technology given their ability to delineate communicative intent of the user through the direct monitoring and analysis of brain activity, avoiding the need for voluntary motor control (McFarland and Wolpaw, 2011; Tabar and Halici, 2016). Brain-computer interface systems employ signal processing algorithms to extract relevant features from acquired brain signals, and then generate classification models to decode intent or brain state information in real-time (McFarland and Wolpaw, 2011). Despite recent advancements in the field, most BCI research to date has focused on typically developing adults with little work done to investigate the suitability of BCIs for children (Moghimi et al., 2013; Orlandi et al., 2021; Karlsson et al., 2022). Further, many BCI systems designed for communication employ interfaces for spelling (e.g., BCI spellers based on the P300 paradigm, Rezeika et al., 2018), which may not be suitable for children with physical disabilities and concomitant delays in language and cognitive development.

Alternatively, emotions underlie many of our basic needs, wants, and preferences, and are closely linked to cognition and memory (LeDoux, 2000). If emotional states can be accessed through a BCI, they could provide a pathway to communication that circumvents the need for words or other developed literacy skills. To design a BCI controlled by emotional states, we require a model of the underpinning neurophysiological processes that produce them. Traditionally, emotions have been modeled as discrete entities, each hypothesized to have their own distinct physiological “fingerprint,” such as happiness or anger (Feldman Barrett, 2006). However, a growing body of research has demonstrated a vast amount of variation in these physiological templates across individuals and situations (Hamann, 2012), necessitating a new approach to modeling emotions. The dimensional model of emotion (Russell, 1980) instead postulates that any emotional state falls within two fundamental dimensions: valence, the degree of pleasantness of an emotion, and arousal, the degree of activation of an emotion. While the dimensional model effectively captures the description of an emotional “feeling,” it does not necessarily explain how these states are generated within the brain (Mühl et al., 2014). Appraisal models provide such a hypothesis; that our experience of emotion arises due to systematic “checks” within cognitive networks of our brains to assess and evaluate the relevance, significance, and implications of a perceived stimulus (Scherer, 2005).

Fitting with dimensional and appraisal models, meta-analyses of neuroimaging studies have demonstrated that large functional networks are responsible for emotion processing in the brain, opposed to the simple “one-to-one” mapping of brain structures typically associated with discrete models of emotions (Hamann, 2012). After exposure to a stimulus, limbic neural structures such as the amygdala, the orbitofrontal cortex (OFC), and the anterior insula integrate the incoming sensory information with any associated memories of the stimulus to evaluate its context and assign an emotional value. From the amygdala, signals are distributed to the hypothalamus and brainstem, where autonomic and endocrine response are directed. There are also extensive connections between these limbic structures and parts of the PFC, where higher-level cognitive processes are activated in response to the contextualized emotional stimulus (Davidson, 2002).

It is the activation of the PFC in emotion processing that would allow emotions to be accessed in non-invasive a BCI, as other limbic structures are located too deep inside the skull for superficial detection by portable neuroimaging modalities (Mühl et al., 2014). The PFC is essential for evaluating the emotional significance of a stimulus, interpreting and regulating emotional experience, and directing subsequent behaviors (Dixon et al., 2017; Hiser and Koenigs, 2018). The PFC evaluates the *core affect* of a stimulus—whether it is rewarding or threatening, and if it should therefore be approached or avoided (Feldman Barrett et al., 2007; Dixon et al., 2017; Hiser and Koenigs, 2018). The OFC evaluates incoming sensory information and appraises personal episodic memories related to the stimulus (Feldman Barrett et al., 2007; Dixon et al., 2017). The ventromedial prefrontal cortex (vmPFC) is divided into several substructures that perform a range of functions including directing autonomic changes in physiological arousal, interpreting such autonomic changes to construct subjective “feelings” of emotions, the appraisal of emotional states of others, and directing behavioral actions during emotional responses (Dixon et al., 2017; Hiser and Koenigs, 2018). The lateral prefrontal cortex, including the dorsolateral (dlPFC) and ventrolateral (vlPFC) prefrontal cortex broadly is involved in directing emotional regulation, or the conscious manipulation of an emotional response according to a desired goal (Ochsner et al., 2009; Dixon et al., 2017).

Brain-computer interfaces that seek to detect and interpret emotional states are known as affective BCIs (Mühl et al., 2014). Many existing affective BCI studies have attempted to decode emotional states from electrical brain activity, as measured non-invasively from the scalp using electroencephalography (EEG) (Torres P et al., 2020). These studies vary significantly in methodology, from the way emotions are defined and how emotional responses are elicited, to what features of the EEG are extracted and the algorithms used to optimize and classify these features into categorical emotional states (Mühl et al., 2014). Thus, unsurprisingly, existing affective BCI studies report a wide range of results and levels of performance (classification accuracies ranging from 50% to 90%) (Torres P et al., 2020). The majority of recent studies (77%) have focused on the development of novel classification pipelines, using existing publicly available datasets of EEG recordings generated during emotion-induction tasks to support their research (Torres P et al., 2020). There is also a growing trend to employ deep learning methods like neural networks, although linear discriminant classifiers remain quite popular (Torres P et al., 2020). The generalizability of such methods—use of a static dataset for classifier training and classification models that may obfuscate underlying neurophysiological phenomena—must be verified in future work (McFarland et al., 2017; Torres P et al., 2020).

Functional near-infrared spectroscopy (fNIRS) is an alternative signal acquisition modality that has been increasingly used in the functional mapping of brain activity (Hoshi, 2003), including the imaging of prefrontal cortical activation in emotion processing in adults (Doi et al., 2013) as well as infants (Maria et al., 2018). Because of its relative comfort and robustness to motion artifacts compared to EEG (Hoshi, 2003), fNIRS is particularly suited for exploring BCI solutions for pediatric users. Functional near-infrared spectroscopy uses light in the near-infrared range (~700–1,200 nm) to measure cerebral hemodynamic activity. Near-infrared light is transmitted from a light source (e.g., LED, laser) through the tissues of the head and scalp and is absorbed by oxygenated and deoxygenated hemoglobin (HbO and Hb) in cerebral blood. Unabsorbed light is scattered throughout the cerebral tissue, reflected back out of the head, and measured by detectors on the scalp (Gratton et al., 2003). Change in concentration of oxygenated and deoxygenated hemoglobin (Δ[HbO] and Δ[Hb]) is calculated using a modified version of the Beer-Lambert law (Coyle et al., 2007; Scholkmann et al., 2014), and can be considered an indirect measure of neuronal activation: neuronal activity in a region of the brain increases the metabolic demands of that area, stimulating an increase of blood flow to the brain and resulting in an overall increase in [HbO] and a decrease in [Hb] (Hoshi, 2003; Coyle et al., 2007; Scholkmann et al., 2014). Functional near-infrared spectroscopy is capable of imaging depths of about 1–2 cm into the scalp, penetrating the cortical surface, making it a viable option for investigating the activation of the PFC in emotion processing (Doi et al., 2013; Bendall et al., 2016).

Several studies have investigated fNIRS as a signal acquisition modality for affective BCIs. Tai and Chau (2009) were the first to attempt single-trial classification of emotional state from cerebral hemodynamic activity, and were able to discriminate emotion-induced brain activation from a neutral state with at least 75% accuracy. Hosseini et al. (2011) and Moghimi et al. (2012), differentiated positive and negative emotional states induced by affective images and music excerpts, respectively. Heger et al. (2014) were able to discriminate emotions on the dimensions of both valence and arousal at rates significantly above chance. Yanagisawa and Tsunashima (2015) found that BCI classification accuracy correlated with the intensity of an individual's emotional reactions to stimuli, and Hu et al. (2019) investigated the discrimination of different types of positive emotions. An online interface has also been designed where users could interact with a virtual character through positive emotions (Aranyi et al., 2016) or anger (Aranyi et al., 2015). In a series of studies, Trambaiolli et al. used fNIRS to differentiate emotional states both passively (viewing emotionally salient images) and actively (self-generating emotional memories), both offline (Trambaiolli et al., 2018b) and exploring the effects of online neurofeedback (Trambaiolli et al., 2018a).

All these reviewed studies were conducted with a population of neurotypical adults and all but three were conducted offline, meaning that BCI performance was evaluated retrospectively, without providing immediate feedback of system control, and without evaluating the generalizability of classification pipelines to new, unseen data. In this paper, we describe for the first time the development, training, and testing of a pediatric fNIRS BCI system to predict emotional valence in school-aged children from their cerebral hemodynamic activity in real-time, with the goal of determining the feasibility of an affective BCI as an access pathway to communication for children.

Participants attended four study sessions, the first to collect data for initial training and development of the BCI, then the remainder to evaluate the BCI system online. In each session, participants underwent a series of emotion-induction trials while their hemodynamic activity was measured using fNIRS. The collected hemodynamic signals were labeled and used to train and test a classifier to recognize emotional valence. During the online sessions, the BCI classified emotional states in real-time and participants were presented with visual feedback representing the real-time emotional state predictions. Brain-computer interface performance was evaluated through the percentage of correct classification of emotional states in real-time (accuracy) as well as classifier sensitivity and specificity.

## 2. Methodology

### 2.1. Participants

Ten neurotypical children (three male) between the ages of 8 and 14 years (mean 11.5±1.75 years) were recruited for this study. Participants were screened for any neurological, psychological, cardiovascular, or respiratory conditions, as well as for any history of brain injury or emotional trauma. The study received approval from the respective research ethics boards of Holland Bloorview Kids Rehabilitation Hospital and the University of Toronto, Toronto, Canada. Written consent/assent was obtained from all participants or their parent/guardian.

### 2.2. Instrumentation

**Figure 1:**
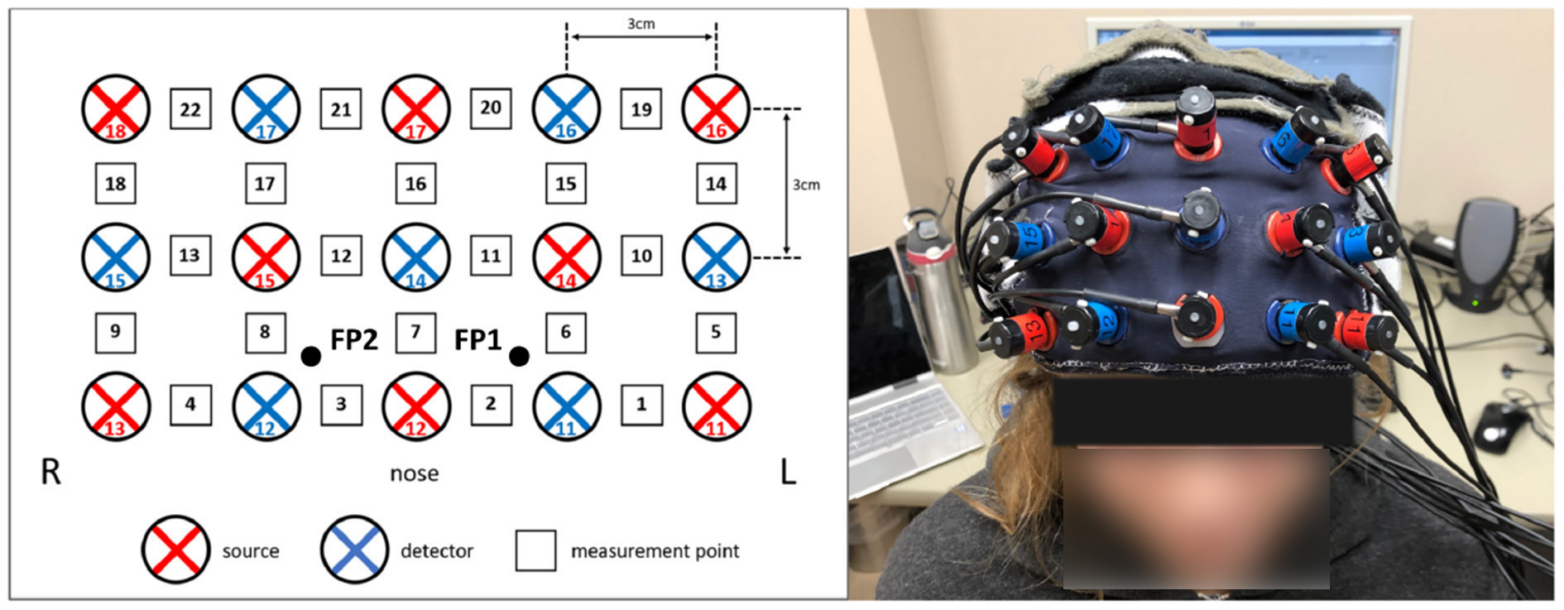
fNIRS optode configuration over the prefrontal cortex. **(Left)** The eight light sources (red) and seven light detectors (blue) were arranged in a 3 × 5 grid over the forehead, resulting in 22 channels (black and white squares). **(Right)** The optodes, mounted in the headpiece and placed over the forehead.

Cerebral hemodynamic activity was measured using the Hitachi ETG-4000 NIRS system (Hitachi Medical Systems, Tokyo, Japan). A 3 × 5 grid of eight light emitters and seven light detectors was secured over the PFC using a custom-made headpiece, with the bottom row of optodes sitting just above the eyebrows and centered at the nose. Detectors 11 and 12 (Figure 1) were approximately aligned with the Fp1 and Fp2 sites of the 10–20 International System of electrode placement and the top row were approximately aligned with the F5–Fz–F6 sites (Schudlo and Chau, 2015). Each emitter and detector were separated by 3 cm, corresponding to a measurement depth of 2–3 cm (Okada et al., 1997; Haeussinger et al., 2011), reaching the cortical surface (Coyle et al., 2004b). This arrangement resulted in 22 integrated channels, as indicated in Figure 1. Data were sampled at 10 Hz.

### 2.3. Experimental protocol

#### 2.3.1. Session structure

**Figure 2:**
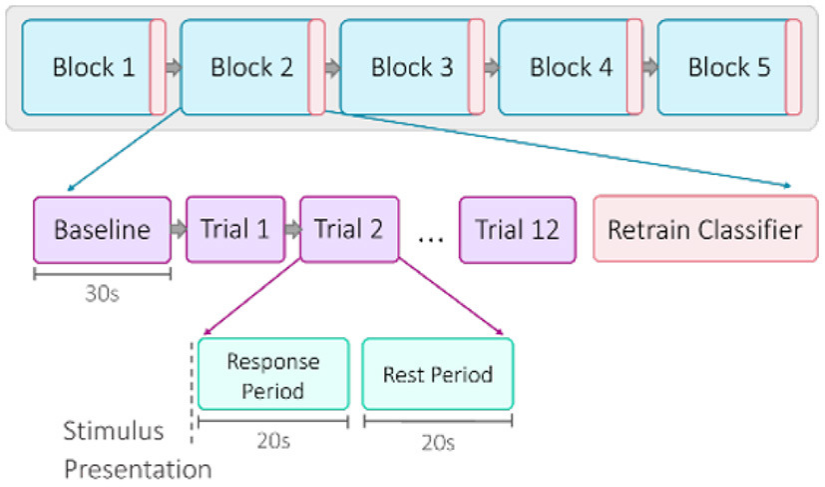
Overview of session structure. Each of the five blocks began with a 30-s baseline recording, followed by 12 trials. A trial was composed of a 20-s response period of stimulus presentation and a 20-s rest period. For the online sessions, the classifier was retrained after each block.

A single session was comprised of five blocks of emotion induction trials (Figure 2). Each block began with a 30-s baseline recording. Then for each trial, a set of emotional stimuli was presented for a 20-s response period (Coyle et al., 2004b). All stimuli used for a single trial were matched for valence and arousal. A prompt on the screen labeled each trial as “positive” or “negative,” confirming for the participants the intended valence. For the initial offline session, the participants were instructed to react naturally to the stimuli. For the online sessions, the participants were instructed to use the provided visual feedback as a guide to help strengthen their emotional response. Each response period was punctuated by a 20-s rest period, allowing hemodynamic activity to return to baseline levels (Schudlo and Chau, 2015; Weyand et al., 2015a). There were 12 trials within one block, for a total of 60 trials (30 positive, 30 negative) in one session. For the online sessions, the classifier was re-trained after each block. When a block was completed, participants would self-select when to proceed, allowing for an optional short break. Each session took approximately 40–50 min to complete.

#### 2.3.2. Affective stimuli

Bimodal stimulation has been shown to enhance brain activation in emotion processing (Baumgartner et al., 2006), so the affective stimulus set included both visual and auditory stimuli presented simultaneously. The visual stimuli consisted of pictures drawn from three standardized databases: the International Affective Pictures System (IAPS), the Geneva Affective Pictures Database (GAPED), and the Open Affective Standardized Image Set (OASIS). These databases are collections of color photographs from a wide range of semantic categories that have been reproducibly rated on their affective quality on scales of valence and arousal (Lang et al., 2008; Dan-Glauser and Scherer, 2011; Kurdi et al., 2017), and reliably evoke emotional responses in children (McManis et al., 2001; Sharp et al., 2006; Hajcak and Dennis, 2009). The selection of pictures was personalized for each participant. The auditory stimuli consisted of 20-s excerpts of music, chosen from modern genres to ensure high saliency for the pediatric population. Excerpts were sampled without lyrics to reduce potential brain activation due to mental singing, and were rated by a music therapist for valence and arousal based on their tempo and mode (Dalla Bella et al., 2001; Nieminen et al., 2011). The stimulus set for a single trial was composed of one 20-s musical excerpt and five affective images, matched for valence and arousal. Each image was displayed for 4-s each, while the music excerpt played. Either a positively- or negatively-valenced set of stimuli was presented for each trial, in a counter-balanced and pseudo-randomized order. For the 20-s rest period, a clip of brown noise was played, and five neutral images were displayed as a control stimulus.

#### 2.3.3. Visual neurofeedback

**Figure 3:**
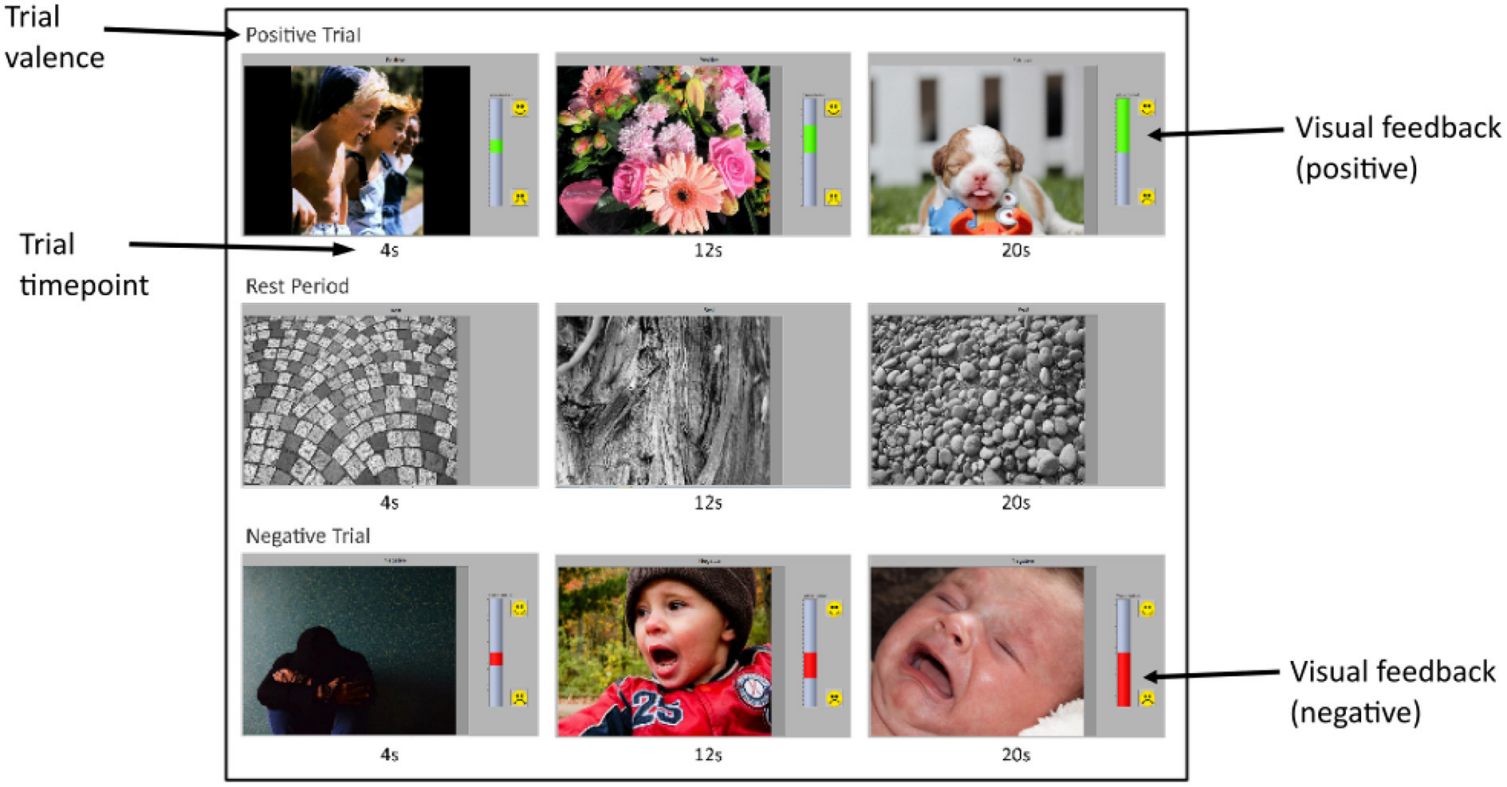
Screenshots of experimental interface. Each row shows three screenshots, taken at 4, 12, and 20-s of an emotion-induction (or rest) trial. The top, middle, and bottom rows show a positive trial, rest period and a negative trial, respectively. Each screenshot shows the stimulus image being presented at that time in the trial, the valence prompt above it, and the feedback bar on the right. Stimulus images were taken from the IAPS (Lang et al., 2008), GAPED (Dan-Glauser and Scherer, 2011), and OASIS (Kurdi et al., 2017) affective image databases. For the positive trial, the feedback bar is green and increasing in height, representing an increasing probability the incoming signal belongs to the positive class as the trial proceeds. Similarly, the feedback bar is red and decreasing in height for the negative trial, indicating an increasing probability that the incoming signal belongs to the negative class as the trial proceeds. The rest trial has no visual feedback.

Visual neurofeedback was provided for the online sessions, in the form of a vertical bar that filled with color according to the predicted valence of the response (Figure 3). Each trial started with the bar at a neutral middle position and would rise in height if the response was predicted to be positive and lower in height if the response was predicted to be negative. Participants were instructed to try to raise or lower the height of this bar as much as possible over the 20-s trial response period, according to the prompted valence of that trial. The BCI would process, analyze, and classify 2-s segments of real-time, incoming data, and update the height of the feedback bar based on the classification output. The specific height of the feedback reflected the probability that the incoming signal segment belonged to either the positive or negative emotional valence class. The BCI would analyze each segment cumulatively; that is, the first classification output would be based on the first two seconds of the response period, the second would be based on the first 4-s, and so on until the classification of the entire 20-s signal.

#### 2.3.4. Questionnaires

At the end of each session, participants answered a short questionnaire about their subjective experience, which included questions on their mood, fatigue, and perceived effort and frustration throughout the session. Participant temperament, which can affect the experience and ability to regulate emotions (Rothbart, 2007), was also assessed, using the parent-reported version of the Early Adolescent Temperament Questionnaire (EATQ), developed by Capaldi and Rothbart (1992) and validated by Muris and Meesters (2009).

### 2.4. Data analysis

#### 2.4.1. Signal processing

Physiological noise sources contaminating the hemodynamic signal include cardiac activity (0.8–1.2 Hz), respiration (0.2–0.4 Hz), and Mayer waves, or fluctuations due to arteriole pulsations (0.1 Hz)(Coyle et al., 2004a; Scholkmann et al., 2014; Naseer and Hong, 2015). These were removed from the signal using a third order type II Chebyshev low-pass infinite impulse response (IIR) filter with a passband of 0–0.1 Hz, a transition band of 0.1–0.5 Hz, and a stopband cut-off frequency of 0.5 Hz, with a ripple of 0.1 dB and a minimum attenuation of 50dB (Schudlo and Chau, 2015; Weyand et al., 2015a). The mean of the 30-s baseline recording for each block was also computed and subtracted from subsequent trials in that block (Rezazadeh Sereshkeh et al., 2019).

#### 2.4.2. Feature extraction and selection

Seven features capturing the morphology of the temporal hemodynamic signal were investigated as potential feature types—mean, slope, moving slope (4-s window, 0.5-s overlap), variance, root mean squared (RMS), skewness, and kurtosis (Naseer and Hong, 2015). These features were calculated over the entire 20-s trial response period, for each of the two chromophores ([HbO] and [Hb]) and each of the 22 channels. Time-frequency feature (i.e., from wavelet decomposition) were also considered, but were found in prior work to offer only marginal value when used in combination with temporal features compared to temporal features alone (Tai and Chau, 2009). Lateral asymmetry features, comparing the difference in the hemodynamic signal between the right and left sides of the PFC, were also investigated but found to not be discriminatory in preliminary study analyses. The seven feature types were tested individually for each participant using the data collected from their first session. The most discriminatory feature for each participant, i.e., the feature generating the highest classification accuracy after 10 iterations of 10-fold cross-validation, was then used to train the classifiers for their upcoming online sessions. Two chromophores and 22 channels yielded a feature set of 44 features. A 44-dimension feature set was considered too large for the amount of data that could be collected within a single session (*n* = 30 each class) (Jain et al., 2000; Kudo and Sklansky, 2000). A sequential forward floating search (SFFS) algorithm was used for feature set dimensionality reduction, selecting the five best channels for each chromophore, resulting in a subset of 10 features. Sequential forward floating search systematically searches through the available features to create an optimal subset, adding the best *l* features and removing the worst *r* features each iteration based on a fitness criterion (Pudil et al., 1994). The Fisher criterion was used as the fitness criterion; refer to Power et al. (2011) and Schudlo and Chau (2014) for more information on this method. Feature selection was performed on the partitioned data—that is, the “test” fold was excluded from feature selection and used only to evaluate classifier performance.

#### 2.4.3. Classification

A linear discriminant analysis classifier (LDA) was used to differentiate the acquired hemodynamic signals based on emotional valence. Linear discriminant analysis is a commonly used classification algorithm for online BCI applications due to its speed and low computational cost (Nicolas-Alonso and Gomez-Gil, 2012). Linear discriminant analysis involves defining a linear decision boundary that separates the data into two classes, maximizing the distance between the class means while minimizing the variance within each class (Nicolas-Alonso and Gomez-Gil, 2012).

#### 2.4.4. Online classifier retraining

**Figure 4:**
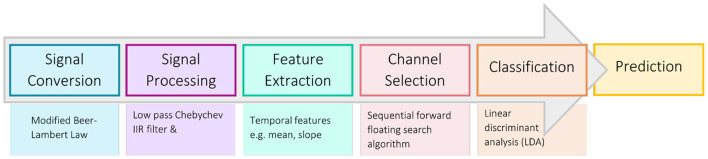
Data analysis pipeline for online sessions.

A classifier was trained for each participant using their most discriminatory feature (chosen from their first session results) and was then used to predict emotional valence in real-time during the online session (Figure 4). This classifier was retrained after each block of trials within each online session, as classifier performance has been shown to improve with the incorporation of same-day training data (Power et al., 2012; Rezazadeh Sereshkeh et al., 2019). Retraining involved running all collected data through the data analysis pipeline, selecting five new “best channels” for each chromophore, and then training a new LDA classifier. After each online session, the classifier was updated to incorporate all existing data from all completed sessions.

## 3. Results

### 3.1. Online session results

**Figure 5:**
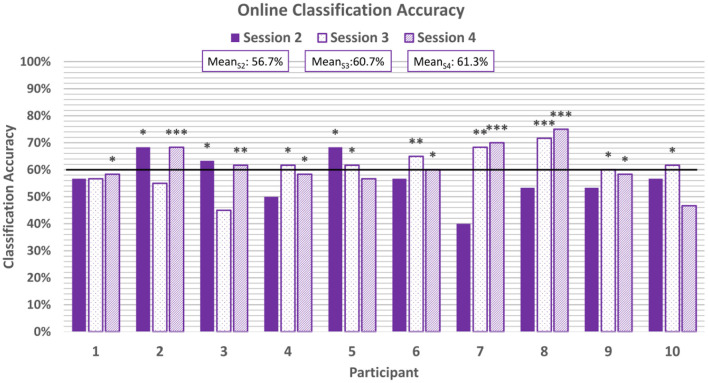
Average classification accuracies for each online session. The horizontal line represents the 95% confidence level threshold for the first online session (based on *n* = 60 emotion-induction trials). Note that the 95% confidence level threshold drops down from 60% to 58% and 57% with each session as the amount of cumulative data increases (Müller-putz et al., 2008; Combrisson and Jerbi, 2015). Accuracies exceeding the upper limit of the 95%, 99%, and 99.9% confidence intervals of chance are marked with *, **, and ***, respectively.

For the online sessions, BCI performance was evaluated based on accuracy of real-time predictions. A correct classification was tallied if the predicted classification matched the valence of the stimuli for that trial. Accuracy was defined as the percentage of correct classifications out of all classifications made. The average classification accuracy for each participant for each online session can be seen in Figure 5 and broken down by block in Table 1. All participants achieved session-wide average accuracies exceeding chance, as estimated according to Müller-putz et al. (2008) and Combrisson and Jerbi (2015), for at least one of the three online sessions, and 8 of the 10 participants exceeded chance level for two of the three online sessions. None of the participants exceeded chance level for all three online sessions.

**Table 1:** Online classification accuracies for each session, broken down by block (B1–B5).

**Block**	**Participant**										
	**P1**	**P2**	**P3**	**P4**	**P5**	**P6**	**P7**	**P8**	**P9**	**P10**	**Avg**
**Session 2**											
B1	41.7	41.7	66.7**	33.3	75.0***	58.3	58.3	33.3	50.0	41.7	50.0
B2	58.3	83.3***	66.7**	83.3***	83.3***	50.0	41.7	50.0	50.0	41.7	60.8*
B3	25.0	66.7**	66.7**	50.0	58.3	58.3	41.7	66.7**	50.0	75.0***	55.8
B4	75.0***	66.7**	66.7**	41.7	58.3	66.7**	25.0	66.7**	75.0***	41.7	58.3
B5	83.3***	83.3***	50.0	41.7	66.7**	50.0	33.3	50.0	41.7	83.3***	58.3
Avg	**56.7**	68.3**	63.3*	**50.0**	68.3**	**56.7**	**40.0**	**53.3**	**53.3**	**56.7**	**56.7** **±16.2**
**Session 3**											
B1	66.7**	58.3*	41.7	50.0	50.0	50.0	41.7	75.0***	75.0***	50.0	55.8
B2	75.0***	41.7	25.0	58.3*	58.3*	100.0***	75.0***	66.7**	75.0***	58.3*	63.3**
B3	41.7	75.0***	58.3*	50.0	58.3*	66.7**	83.3***	75.0***	41.7	66.7**	61.7**
B4	41.7	33.3	50.0	66.7**	58.3*	33.3	66.7*	58.3*	75.0***	75.0***	55.8
B5	58.3*	66.7**	50.0	83.3***	83.3***	75.0***	75.0***	83.3***	33.3	58.3*	66.7**
Avg	**56.7**	**55.0**	**45.0**	61.7**	61.7**	65.0**	68.3***	71.7***	60.0*	61.7**	60.7* **±16.1**
**Session 4**											
B1	41.7	66.7**	75.0***	75.0***	41.7	33.3	58.3*	100.0***	58.3*	58.3*	61.1*
B2	66.7**	66.7**	75.0***	33.3	50.0	66.7**	83.3***	83.3***	66.7**	50.0	64.2**
B3	50.0	66.7**	91.7***	83.3***	66.7**	58.3*	58.3*	66.7**	33.3	50.0	62.5**
B4	66.7**	66.7**	16.7	50.0	41.7	66.7**	75.0**	58.3*	66.7**	58.3*	56.7
B5	66.7**	75.0***	50.0	50.0	83.3***	75.0***	75.0***	66.7**	66.7**	16.7	67.6***
Avg	58.3*	68.3***	61.7***	58.3*	**56.7**	60.0*	70.0***	75.0***	58.3*	**46.7**	61.3** **±17.2**

Entire session averages are bolded. Accuracies exceeding the upper limit of the 95%, 99%, and 99.9% confidence intervals of chance are marked with *, **, and ***, respectively.

BCI performance tended to improve throughout a single session: on average, the last block of each session was more accurate than the first block of that session (e.g., *mean*_*S*2, *B*1_ = 55.8% and *mean*_*S*2, *B*5_ = 66.7%). However, these differences were not found to be significant based on a two-tail t-test. As more same-day data were accumulated and used to retrain the classifier, it is expected that accuracy will improve (Power et al., 2012). In the present study, session length was limited to five blocks to prevent fatigue among the pediatric participants.

There was also a trend of improvement in performance across the three online sessions (*mean*_*S*2_ = 56.7%, *mean*_*S*3_ = 60.7%, *mean*_*S*4_ = 61.3%). Only 30% of participants exceeded chance-level accuracies for the first online session, while 70% and 80% participants exceeded chance for the second and third online sessions, respectively. A one-way ANOVA of the average classification accuracies did not reveal a significant difference across the sessions (*p* = 0.38, Shapiro-Wilk test for normality). This could be attributed to the fact that some participants exhibited anomalous performance in one of their sessions (e.g., Participants 2 and 3), deviating from the overall trend of improvement.

**Figure 6:**
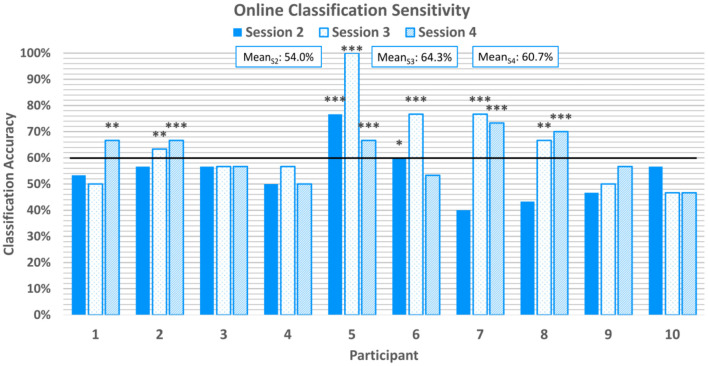
Average classification sensitivity for each online session. Sensitivity refers to the correctly classified positive emotion-induction trials out of all positive emotion-induction trials. The horizontal line again represents the 95% confidence level threshold for the first online session. Sensitivities exceeding the upper limit of the 95%, 99%, and 99.9% confidence intervals of chance are marked with *, **, and ***, respectively.

Online classification sensitivity, which refers to the correct classification of emotion-induction trials with positive valence, averaged at 59.7%±12.7% across all participants and online sessions. Sensitivity scores can be found in Figure 6. 20% of participants achieved above-chance sensitivity scores in the first online session, but 50% achieved above-chance sensitivity scores in the second and third online sessions. With the exception of P6, all participants who achieved above-chance sensitivity scores in the first or second online session also achieved above-chance scores in the third online session.

**Figure 7:**
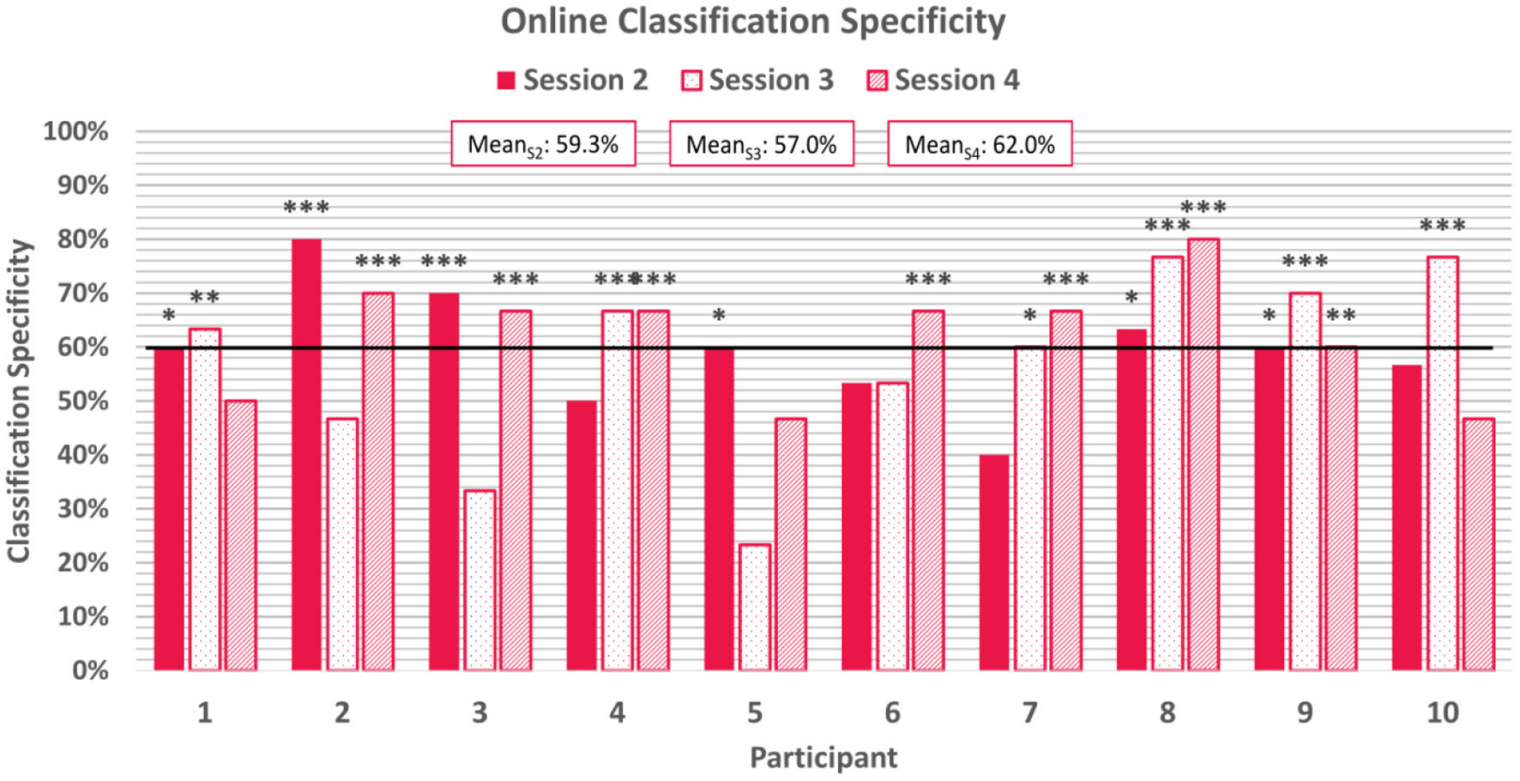
Average classification specificity for each online session. Specificity refers to the correctly classified negative emotion-induction trials out of all negative emotion-induction trials. The horizontal line again represents the 95% confidence level threshold for the first online session. Sensitivities exceeding the upper limit of the 95%, 99%, and 99.9% confidence intervals of chance are marked with *, **, and ***, respectively.

Average online classification specificity, which refers to the correct classification of negatively-valenced emotion induction trials, was observed to be 59.4% ± 13.4% across participants and sessions. 60% of participants achieved above-chance specificity scores for the first two online sessions, although were not all the same participants, and 70% of participants achieved above chance scores for the final online session. Two participants achieved above-chance scores for all three online session. Specificity scores can be found in Figure 7.

**Figure 8:**
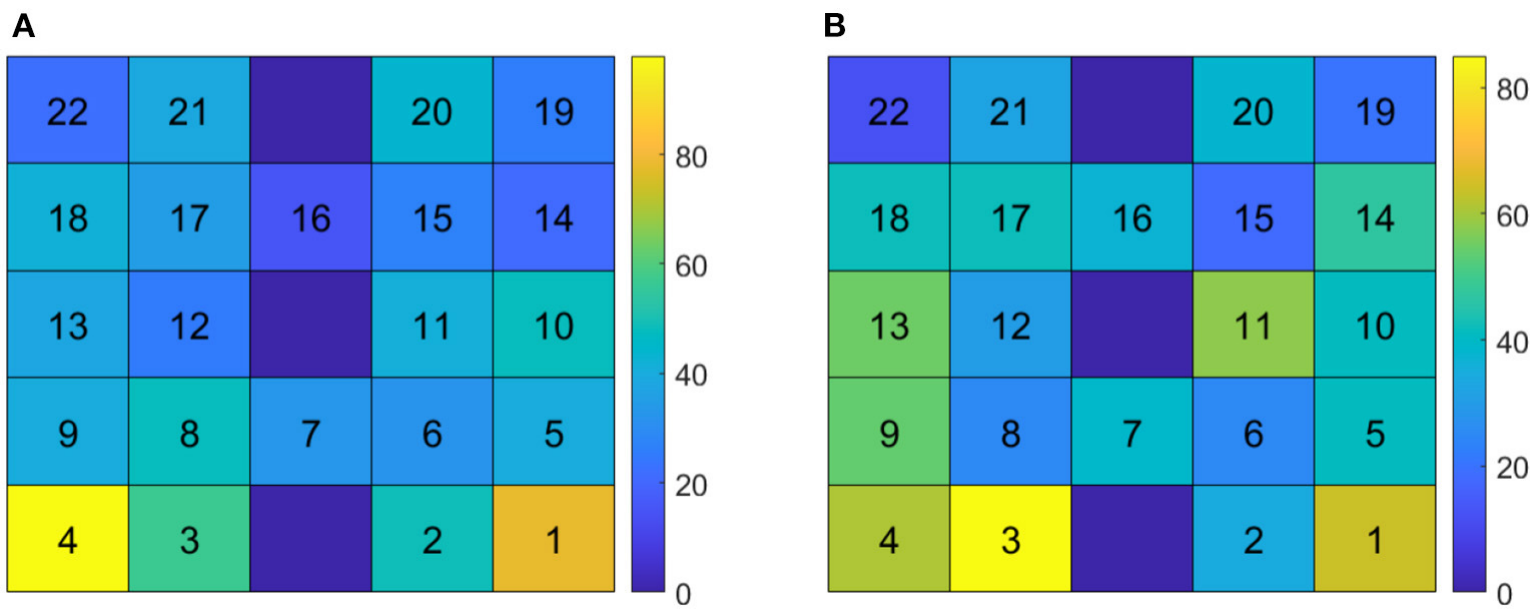
Channel selection frequency across participants and sessions. **(A)** Channel selection frequency for Δ[Hb] features. **(B)** Channel selection frequency for Δ[HbO] features. The bottom row corresponds to channels directly over the nose; channels 2 and 3 correspond approximately with Fp1 and Fp2 of the 10/20 EEG standard montage.

Total frequency of each channel selected for use after the feature selection step of the classification pipeline is shown in Figure 8, across all participants and sessions. Discriminatory channels varied across participants but were selected bilaterally, and most frequently selected over areas corresponding approximately to the ventrolateral and dorsolateral prefrontal cortices.

### 3.2. Interparticipant variability

#### 3.2.1. Hemodynamic response

**Figure 9:**
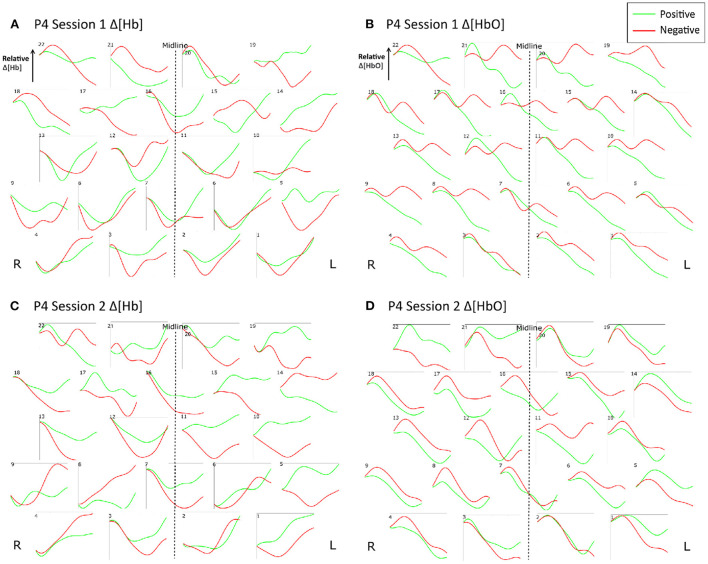
Trial-averaged hemodynamic response functions (Δ[HbO] and Δ[Hb]) for P4 for Sessions 1 and 2. **(A)** Δ[HbO] for Session 1, **(B)** Δ[HbO] for Session 2, **(C)** Δ[Hb] for Session 1, and **(D)** Δ[Hb] for Session 2. The response function for each channel is shown mapped according to its position over the forehead. The lighter shade (green) indicates the average of all positive trials, and the darker shade (red) indicates the average of all negative trials from the respective session.

**Figure 10:**
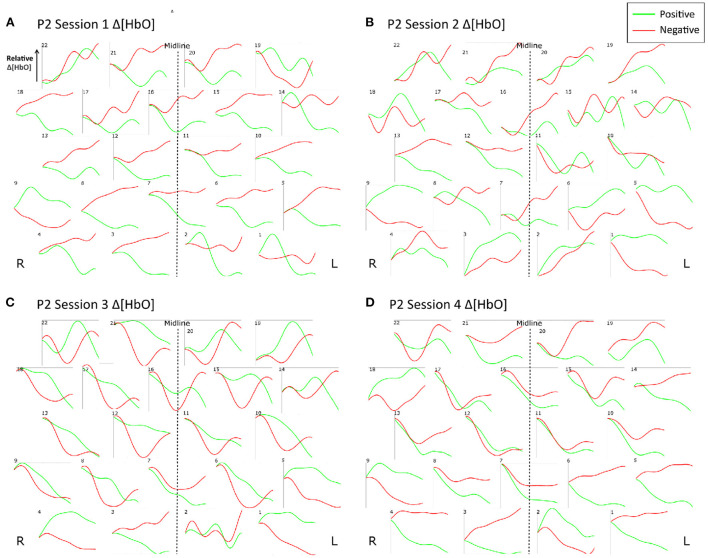
Trial-averaged hemodynamic response functions (Δ[HbO]) for P2 for all four sessions. **(A)** Δ[HbO] for Session 1, **(B)** Δ[HbO] for Session 2, **(C)** Δ[HbO] for Session 3, and **(D)** Δ[HbO] for Session 4. The response function for each channel is shown mapped according to its position over the forehead. The lighter shade (green) indicates the average of all positive trials, and the darker shade (red) indicates the average of all negative trials from the respective session.

Figure 9 shows the trial-averaged hemodynamic response function (HRF) for *both* Δ*[HbO] and* Δ *[Hb]* for a single participant, P4, for the *first two sessions*. The response signal from each measurement channel is shown mapped according to its position over the forehead. A clear distinction can be seen between the positive and negative response for both chromophores in both sessions. However, the responses vary across different session days. Figure 10 shows the trial-averaged HRFs for *just* Δ*[HbO]* for P2 for *all four sessions*. Once again, variability in the response can be seen across the different sessions. Interestingly, the HRF for session 3 appears visually dissimilar from sessions 1 and 2, which could possibly account for the reduced classification accuracy achieved in that session. Similar patterns were seen for the other participants as well. Further analysis is needed to quantify this intersession variability in the hemodynamic response.

#### 3.2.2. Mental state

At the end of each session, participants were asked to complete a questionnaire assessing their mental state before, during, and after the session in terms of alertness/fatigue, mood, effort, and frustration. At the group level, there were no significant correlations between these factors and BCI performance. However, trends linking mental state and BCI performance on different sessions days were observed individually. For example, P4 experienced the greatest amount of fatigue during their session with their poorest BCI performance and experienced the least fatigue during their highest-scoring session, suggesting that their level of fatigue may have contributed to their ability to use the BCI effectively. Some work has already been done to investigate the effects of mood and fatigue on BCI performance (Myrden and Chau, 2015), this should be extended to pediatric populations as well.

#### 3.2.3. Age

**Figure 11:**
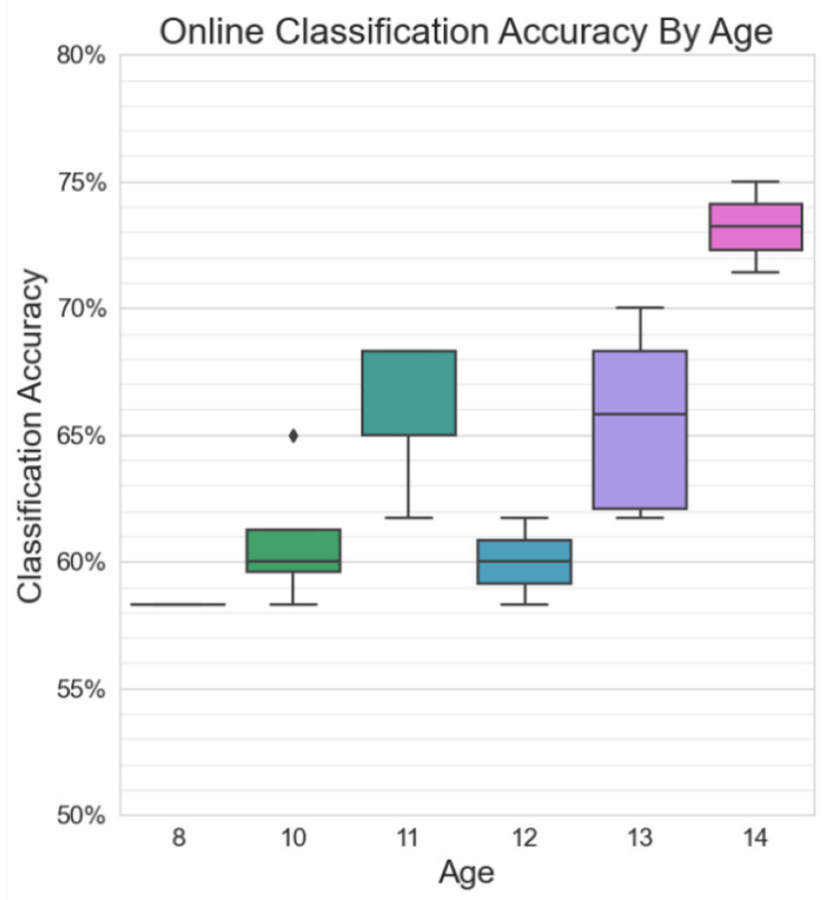
Classification accuracies in excess of chance for online sessions, grouped by age. There is a moderate significant positive correlation between age and BCI performance (*r* = 0.63, *p* = 0.03).

Figure 11 shows greater-than-chance level online classification accuracies grouped by age. A significant positive correlation was found between online BCI performance and age for statistically accurate online sessions (*r* = 0.63, *p* = 0.03). Three of the highest performers (P5, P7, and P8) were the three oldest participants, all at least 13 years old. The two lowest performers (P1 and P9) were two of the youngest participants, at ages 8 and 10.

#### 3.2.4. Temperament

The parent-reported Early Adolescent Temperament Questionnaire (EATQ) assessed participants' temperament along ten different dimensions: activation control, affiliation, aggression, attention, depressive mood, fear, frustration, inhibitory control, shyness, and surgency. Each question on the EATQ was answered with a five-point Likert scale and was categorized under one of the 10 dimensions. Mean scores for each dimension were calculated by averaging the responses for all the questions pertaining to that dimension, resulting in a score from 1 to 5 for each of the 10 dimensions. These scores were compared to online BCI performance (classification accuracy, sensitivity, and specificity) for each participant. No significant correlations were found between BCI performance and any of the temperament measures.

### 3.3. Offline cross-validated classification results

In addition to the real-time, online classification accuracies, 10 iterations of 10-fold cross-validation were used to evaluate BCI performance offline. The resulting average classification accuracies can be found in Table 2. Thirty-eight of the forty sessions exceeded the 95% confidence level of chance of 65% (Müller-putz et al., 2008; Combrisson and Jerbi, 2015). Thirty-five of the forty exceeded the 99% confidence level, and fifteen of the forty sessions exceeded the 99.9% confidence level. The average offline classification accuracies across all participants was 78.4%±7.8%, 77.4%±3.7%, 76.7%±9.5%, and 76.9%±7.7% for sessions 1–4, respectively.

**Table 2:** Average offline classification accuracies for all four sessions.

	**Participant**										
**Session**	**P1**	**P2**	**P3**	**P4**	**P5**	**P6**	**P7**	**P8**	**P9**	**P10**	**Avg**
1	74.3**	90.2***	73.3**	74.2**	92.7***	80.5***	71.7**	82.8***	71.0**	73.3**	78.4**
2	75.7**	80.5***	78.2**	71.7**	78.8**	73.2**	80.0***	74.3**	77.7**	83.8***	77.4**
3	62.3	80.0***	60.0	72.5**	91.3***	83.3***	78.5**	79.3**	81.2***	78.7**	76.7**
4	69.2*	85.5***	68.5*	78.5**	78.5**	79.2**	82.8***	88.2***	72.8**	65.3*	76.9**
Avg	70.4**	84.0***	70.0**	74.2**	85.3***	79.0**	78.3**	81.2***	75.7**	75.3**	77.3**

Accuracies were calculated from 10 iterations of 10-fold cross validation. Accuracies exceeding the upper limit of the 95%, 99%, and 99.9% confidence intervals of chance are marked with *, **, and ***, respectively.

## 4. Discussion

### 4.1. Feasibility of a pediatric fNIRS affective BCI

The results of this study suggest that it is possible to discriminate emotional valence from hemodynamic activity in children based on a bimodal emotion-induction task. Out of all 40 sessions, there were only two where chance level classification accuracies were not achieved in an offline 10-fold cross-validation analysis. Average cross-validated classification accuracies for each participant across their four sessions ranged from 70.0% to 85.3% (mean 77.3%±7.2%). This study is the first, to the authors' knowledge, to investigate an affective BCI for children using fNIRS, and the classification accuracies achieved in this study are similar to those reported in affective fNIRS-based BCI studies conducted in adult populations. The average cross-validated classification accuracies are comparable to studies with analogous classification tasks (i.e., discriminating positive and negative valence), such as Hosseini et al. (2011) (mean accuracy 70.6%) and Moghimi et al. (2012) (mean accuracy 71.9%). Tai and Chau (2009) and Trambaiolli et al. (2018b) achieved higher classification accuracies (mean 84.6% emotion vs. neutral, and 89.9% positive vs. neutral, 81.5% negative vs. neutral, respectively), although the classification task in these studies was between a presumably more distinct set of classes—i.e., emotional vs. a neutral/rest state, rather than positive vs. negative. Heger et al. (2014) reported lower classification accuracies, but were investigating the multi-class discrimination of emotional arousal in addition to valence.

The study results also suggest that emotion prediction in children in real-time may also be feasible using an fNIRS-BCI. Every participant in this study had at least one session where they were able to exceed chance-level classification accuracies with online emotion prediction, and 8 of the 10 participants exceeded chance level for two of their three online sessions. A trend of improvement in performance across online sessions was observed, which could be due to enhanced classifier performance from an increasing amount of training data or participants becoming more familiar with emotional regulation. Limited related work has explored online, real-time classification of emotional states using fNIRS in adults. In Trambaiolli et al. (2018a), participants self-regulated their emotional state using real-time BCI classification output as visual feedback, and a median of 70% classification accuracy distinguishing positive emotional states from neutral was observed with just over half of their participants achieving this benchmark. Aranyi et al. mapped affective features in the fNIRS signal to facial expressions of a virtual character in an affective BCI-neurofeedback study, and observed that participants were able to successfully regulate their hemodynamic activity at a success rate of about 50% for positive (Aranyi et al., 2016) and 67% for negative emotions (Aranyi et al., 2015).

The ability to differentiate positive and negative emotional states from hemodynamic activity in a BCI suggests underlying neurophysiological differences in the processing of emotional valence. These differences have historically been attributed to two functional neural systems—the approach system, which primes an individual for approach, attachment or appetitive behaviors, and the withdrawal system, which primes an individual for avoidance, flight or defense (Davidson, 2002). Stimuli that evoke negative emotions such as fear or anxiety activate brain regions of the withdrawal system, facilitating avoidance, while stimuli that produce positive emotions such as happiness or excitement recruit the approach system, facilitating advancement toward the rewarding stimulus (Davidson, 2002). Differential lateral activation has been observed in the PFC, with right PFC activation associated with unpleasant emotional stimuli or the withdrawal system, and left PFC activation associated with pleasant emotional stimuli or the approach system (Davidson et al., 1990). This is known as the *valence asymmetry* hypothesis and has been observed in activation of the PFC through a variety of imaging modalities including EEG (Davidson, 1992), fMRI (Canli et al., 1998; Herrington et al., 2005), and fNIRS (Morinaga et al., 2007; Marumo et al., 2009; Tuscan et al., 2013; Balconi et al., 2015).

In the current study, features of lateral asymmetry were not found to be discriminatory for use in an online affective BCI classification task. Despite the historical popularity of the valence asymmetry hypothesis, there is also a large body of work that has failed to support or found only partial support for the hypothesis, observing overlap in lateral activation of the PFC in response to positively and negatively valenced stimuli (Herrmann et al., 2003; Lewis et al., 2007; Yang et al., 2007; Colibazzi et al., 2010; Hoshi et al., 2011; Moghimi et al., 2012). It is now more widely accepted that the approach-withdrawal network may be driven more broadly by goal-directed motivations rather than valence alone (Carver and Harmon-Jones, 2009; Berkman and Lieberman, 2010). In this hypothesis, approach/withdrawal behaviors can be stimulated either by lower-level stimulus appraisal (i.e., the desire to move away from something unpleasant, like a disgusting smell) or higher-level, top-down goal pursuit (i.e., avoiding a pleasant stimulus like the smell of freshly baked cookies because it contradicts ones' desire to lose weight) (Berkman and Lieberman, 2010). Anger is an example of an emotion that may have approach-related motivations despite being negatively valent and has been found to follow asymmetrical patterns of lateral activation in the PFC consistent with the approach-withdrawal hypothesis (Carver and Harmon-Jones, 2009; Berkman and Lieberman, 2010). It is possible that in the current study, had the emotion induction stimuli been controlled for *action* (i.e., the desire to approach/withdraw) rather than valence alone, better performance with the BCI could have been achieved.

Alternatively, the ability to classify emotional states in real-time could be a result of learning to self-modulate neural activity in response to the provided visual neurofeedback (Sitaram et al., 2017). Neurofeedback training has been investigated to ameliorate particular behaviors or “rewire” certain pathological networks, and has shown to produce lasting functional changes in the brain using EEG (Thibault et al., 2016), fMRI (Paret et al., 2019), and fNIRS (Kohl et al., 2020). fMRI neurofeedback training for emotional regulation has been, in particular, quite broadly researched and has been shown to promote successful self-regulation of activation in the amygdala, anterior insula, anterior cingulate cortex (ACC), and the PFC (Linhartová et al., 2019). Neurofeedback training has also been shown to be feasible in children, including work by Cohen Kadosh et al. where children and adolescents were successfully able to upregulate and downregulate activation in the amygdala using fMRI-based neurofeedback, mitigating symptoms of anxiety (Cohen Kadosh et al., 2016; Lipp and Cohen Kadosh, 2020; Zich et al., 2020). In BCI studies, closed-loop neurofeedback training has been used to help participants produce more reproducible brain activity and thus achieve better control with the BCI (Perdikis and Millán, 2020; Benaroch et al., 2021; Roc et al., 2021). Further, Weyand et al. (2015b) showed that participants could eventually “wean off” neurofeedback, gradually learning to self-modulate their hemodynamic activity in an fNIRS-based BCI in the absence of any visual feedback. Future investigation would be needed to see if with additional emotional regulation and neurofeedback training, better performance could be achieved in an online, affective, fNIRS-based BCI.

### 4.2. Interparticipant variability

Brain-computer interface performance varied considerably across participants, with some achieving online accuracies as high as 75%, others struggling to surpass the chance level threshold. Average sensitivity and specificity scores suggest that some participants may have been better at controlling positively valenced emotions, while others were better at modulating negative valenced emotions. This variability in performance was also supported by variability observed in individual participant hemodynamic response functions as well as in the selection of discriminatory channels chosen for each participant by the feature selection algorithm. Such interparticipant variability is not uncommon—individual differences in performance have been well-documented in research on both neurofeedback training (Alkoby et al., 2018; Kadosh and Staunton, 2019; Weber et al., 2020) and endogenous BCI paradigms (i.e., modulation of sensorimotor rhythms through motor imagery) (Saha and Baumert, 2020; Zhang et al., 2020). The term BCI “inefficacy” has been coined to describe users who are unable to master control over their brain activity (Alkoby et al., 2018), and has been attributed to a variety of psychological, neurophysiological, structural, and protocol-related factors.

Motivation is one psychological trait that has been shown to affect individual performance in neurofeedback/BCI paradigms (Kadosh and Staunton, 2019), with low motivational incongruence (mismatch between goals and perceived achievement) (Diaz Hernandez et al., 2018), high mastery confidence and low incompetence fear (Nijboer et al., 2010), and increased sensitivity to reward (Alkoby et al., 2018) shown to correlate with better performance. Characteristics of resting state or baseline neurophysiological activity have also been shown to correlate with neurofeedback/BCI performance; for example, resting state power of the sensorimotor rhythm frequency band (12–15 Hz) predicted performance in both neurofeedback conditioning training (Reichert et al., 2015) and motor imagery-based BCI (Blankertz et al., 2010; Suk et al., 2014). Structurally, volume of gray and white matter in various cortical regions (Kasahara et al., 2015; Ninaus et al., 2015) may also be associated with performance. Finally, protocol-related factors such as mental modulation or motor imagery strategy used (Kober et al., 2013), amount of training (Esteves et al., 2021), and saliency and design of feedback (Lotte et al., 2013) can also impact performance across individuals. The impact of these factors has yet to be explored and validated for pediatric populations, yet the added variability and complexity of the developing brain will likely further contribute to individual variations in performance, emphasizing the need for personalized training protocols, feedback design, and BCI solutions for pediatric users.

For affective BCIs in particular, differences in temperament across participants should also be considered, as temperament is closely linked with ability to self-regulate emotions (Rothbart, 2007; Bates et al., 2008). Temperament has been linked to differences in recruitment of the approach and withdrawal systems; it has been found that individuals with higher withdrawal or avoidant temperaments respond more strongly to negatively valenced words in an emotional Stroop task, while individuals with higher approach temperaments responded more strongly to positively valenced words in the same task (Mauer and Borkenau, 2007). Further, individuals with higher approach temperaments experience greater physiological changes in response to affective images than those with avoidant temperaments (Yoshino et al., 2005). Effortful control in particular is a temperamental measure that describes capacity and willingness to direct attention and behaviors in order to achieve desired goals, even in the face of averse or unpleasant stimulus (Rothbart et al., 2003). Stadler et al. (2007) observed that children with low levels of effortful control have reduced activation in the ACC—a crucial component of the brain's emotional networks with extensive connections to the PFC—when viewing affective images. In the current study, differences in temperament, as measured by the EATQ, were not found to be correlated with BCI performance. However, due to the lower power of this study, future work is needed to more deeply investigate correlations between individual differences in temperament, emotional self-regulation, and affective BCI performance.

Finally, some affective images used in the stimulus set could have elicited empathetic responses in the participants (i.e., images of children crying). Thus, individual differences in the ability to experience empathy could also contribute to the variability observed in performance. There is some evidence suggesting that predisposition to different types of empathy leads to different patterns of activation in the PFC during emotion processing (Light et al., 2009). However, further research is needed to formalize links between empathetic predisposition, neurophysiological patterns of prefrontal activation, and affective BCI performance.

### 4.3. BCI performance and age

A small significant positive correlation (*r* = 0.63, *p* = 0.03) was found between BCI performance and age, with the three best performers all over 13 years of age. The age of the participants in this study ranged from 8 to 14 years, a period over which considerable emotional, neurophysiological, and cognitive development occurs. Throughout mid-childhood, ongoing developments in cognition, language, and social participation facilitate an increase in emotional awareness in the self and others. Children at this age begin to employ a variety of emotional self-regulation strategies in a variety of contexts, such as re-directing attention from emotionally salient stimuli, re-framing the meaning behind such stimuli, and managing physiological responses to emotional arousal (Thompson, 1991). These strategies continue to mature with adolescence as a more sophisticated sense of self is established and a more nuanced understanding of emotional experiences of others is developed (Thompson, 1991). The temperamental measure of effortful control also plays a key role in the ability to execute emotional self-regulation strategies (Eisenberg et al., 2011), and has also been shown to increase throughout adolescence (Atherton et al., 2020). It is possible that older participants in this study were better able to execute emotional self-regulation strategies to up-regulate or down-regulate their emotional responses and thus achieve better control of the BCI.

Interoception, the perception of one's internal bodily state, is also closely linked with emotional self-regulation ability, with higher interoceptive awareness associated with increased success in emotional regulation and reappraisal (Füstös et al., 2013). Interoceptive sensitivity develops throughout childhood and adolescence (Murphy et al., 2017) and developmental trajectories have been observed in the activation of brain regions implicated in interoception, including the insula (where bottom-up processing of sensory information is integrated with top-down contextual processing) (Li et al., 2017) and the PFC (Klabunde et al., 2019). Differences in interoceptive sensitivity across childhood and adolescence could have led to increasing success with emotional self-regulation and contributed to the trend in improvement in affective BCI performance seen with age in the current study.

Development of temperament, interoception, and emotional self-regulation are supported by the functional neural development of the cortex throughout childhood. The PFC, which directs behaviors required for emotional self-regulation, begins to develop early in childhood but continues to mature throughout adolescence (Tsujimoto, 2008). This maturation process involves a reduction in neuronal density, synaptogenesis, branching, and increased myelination, supporting the specialization of the PFC into functional networks that can carry out the complex cognitive tasks required for a comprehensive awareness and understanding of emotion (Casey et al., 2005; Tsujimoto, 2008). It is possible that the more developed prefrontal cortices of the older participants evoked more reliable and distinct patterns of hemodynamic activity in response to affective stimuli, or were better able to maintain their focus over the course of the session than the younger participants, allowing them to achieve more accurate control of the BCI (Myrden and Chau, 2015).

### 4.4. Intersession (intra-participant) variability

Across the three online sessions, considerable variation was seen within each participant's BCI performance. For example, P2 achieved accuracies of 68% in sessions 2 and 4 but only 55% in session 3. This variability was also reflected in a visual inspection of the signal morphology of the HRFs across different session days. Furthermore, online BCI performance, using classifiers trained on previous session data, was poorer than offline BCI performance where classifier models were based on single-session data. This could have been due in part to this intersession variability.

Variability in the hemodynamic response across different days is a known challenge for fNIRS-BCIs. Holper et al. (2012) found that a greater amount of intersession variability negatively impacted BCI performance in a motor imagery task. Power et al. (2012) found that the hemodynamic response varied across different days in a mental arithmetic task, and Moghimi et al. (2015) observed variability in the hemodynamic response during repeated exposures to musical stimulus. This variability has been attributed to participant-related factors such as changes in fatigue, attention, mood, motivation; physiological factors such as changes in underlying baseline neural activity and basal metabolic rate; environmental factors such as distractions; and to instrumentation-related factors such as deviations in optode placement and calibration (Orihuela-Espina et al., 2010; Power et al., 2012; Myrden and Chau, 2015).

Psychological factors may have also contributed to intra-participant variation. Fatigue, a poor mood or sense of frustration, decreased motivation and attention, could have led to poorer task performance and lower accuracy, which in turn may have worsened mood and led to even more fatigue and frustration as participants struggled to control the BCI. Literature on mechanisms of learning indicates that while some level of challenge can lead to effective problem solving, too much frustration can eventually lead to complete disengagement from a task (D'Mello and Graesser, 2012).

### 4.5. Comparing online and offline BCI performance

Offline cross-validated classification accuracies from analyzing BCI performance for each session *post-hoc* were consistently higher than the real-time, online classification accuracies. This suggests that the classifiers used for real-time prediction had difficulty generalizing to new, unlabeled data (Jain et al., 2000; McFarland and Wolpaw, 2011). The online classifiers, trained on previous session data, could have been limited by intersession variability of the hemodynamic response. Collecting more same-day data for classifier re-training, is known to mitigate the effects of intersession hemodynamic variations (Rezazadeh Sereshkeh et al., 2019).

There were likely samples within the collected data set that were accompanied by shifts in attention, participant movement, distractions, fatigue, or blunted emotional reactions. Thus, higher online classification accuracies might have been achieved with an optimized subset of training data. Subset selection can also minimize computational costs in machine learning problems with large data sets (Mourad et al., 2017).

### 4.6. Study limitations

1. *Modest data quality and quantity:* Children have lower attention spans and fatigue more easily than adults (Plude et al., 1994). As such, the experimental sessions were deliberately abbreviated as much as possible, thereby limiting the amount of data collectable in a single session. Differences in attention were apparent between younger and older participants; the younger participants had more difficulty remaining still and required more frequent breaks. This restlessness likely augmented the risk of artifacts in the acquired hemodynamic signal due to optode-scalp decoupling. Wavering attention likely also contributed to inconsistency of hemodynamic response. Further, the small sample size of this study reduced the ability to investigate the impact of group level differences in age and temperament on BCI performance.
2. *Suboptimal sensor-skin interface:* While fNIRS is relatively robust to motion artifacts (Orihuela-Espina et al., 2010), we could not arbitrarily increase optode-scalp coupling as the children could only tolerate a modest level of probe-on-scalp pressure. In some instances, coupling may have thus been suboptimal. The signal-to-noise ratio of the hemodynamic signal is known to depend, in part, on hair color and density, as well as skin pigmentation (Scholkmann et al., 2014), factors that were not controlled in our sample.
3. *Slow system response:* The hemodynamic response is inherently sluggish, with a post-stimulus peak at about 5–8-s (Coyle et al., 2004b). The long observation window (20 s) deployed in this study limited the practical real-time sensitivity of the BCI. Recent work has suggested that a 5-s stimulation interval was optimal for classification in a sensorimotor fNIRS-BCI task (Afzal Khan and Hong, 2021). Similar investigations could identify an optimal stimulation and response window for affective fNIRS-BCI tasks. Future research may also consider the initial dip in oxygenated hemoglobin concentration as a discriminating cue (Hong and Zafar, 2018).

## 5. Conclusion

Above-chance, online binary differentiation between prefrontal cerebral hemodynamic responses evoked by visual-aural affective stimuli was feasible in a sample of 10 school-aged typically developing children. Classification accuracies from retrospective offline analyses were comparable to those reported in adult affective fNIRS-BCI studies. Brain-computer interface performance was positively correlated with participant age. High variability was observed across sessions, likely due to a combination of physiological, environmental, instrumental and psychological factors. A bimodal affective fNIRS-BCI holds promise as an access modality for school-aged children but longitudinal training and evaluation is necessary in future research to ascertain its practical potential.

This study was the first of its kind to investigate an affective fNIRS-BCI for a pediatric population. It is also one of few studies to conduct real-time prediction of affective state from hemodynamic activity. A BCI that can accurately predict emotional valence could be used to detect emotional states in children with physical disabilities who have had limited success with existing assistive technologies for communication. The binary detection of emotional valence could be used to express feelings, preference or even affirmative/negative responses to questions, without the need for words or other developed language abilities. With access to communication, these children can engage within their communities, learn how to advocate for themselves, gain independence, and overall improve their quality of life.

## Data availability statement

The raw data supporting the conclusions of this article will be made available by the authors, without undue reservation.

## Ethics statement

The studies involving human participants were reviewed and approved by Holland Bloorview Research Ethics Board University of Toronto Research Ethics Board. Written informed consent to participate in this study was provided by the participants' legal guardian/next of kin.

## Author contributions

EF conceptualized and designed the study, collected the data, conducted analyses, and drafted and revised the initial manuscript. SO assisted with data analyses and revised the manuscript. TC oversaw study design, data analyses, and reviewed and revised the manuscript. All authors contributed to the article and approved the submitted version.

## Funding

This study was supported by the Holland Bloorview Kids Rehabilitation Hospital Foundation, and the Kimel Family Foundation.

## Conflict of interest

The authors declare that the research was conducted in the absence of any commercial or financial relationships that could be construed as a potential conflict of interest.

## Publisher's note

All claims expressed in this article are solely those of the authors and do not necessarily represent those of their affiliated organizations, or those of the publisher, the editors and the reviewers. Any product that may be evaluated in this article, or claim that may be made by its manufacturer, is not guaranteed or endorsed by the publisher.
